# Appointment of Dentists in New York Hospitals

**Published:** 1902-06

**Authors:** W. C. Deane


### APPOINTMENT OF DENTISTS IN NEW YORK HOSPITALS.

TO THE EDITOR:

SIR,—In the March number of the INTERNATIONAL DENTAL JOURNAL, regarding the discussion of Dr. Charles F. Allen's paper, "What should be the Relation of our Government to the Dental Profession?" I would like to say that during the discussion Dr. R. H. M. Dawbarn stated, on page 186, "I am glad to say that your branch of our profession is at last being recognized in hospital work, and now in at least two of our greatest hospitals (the New York City and the Kings County) dental surgeons are members of the staff. . . . At the present time these dental surgeons in the New York Hospital occupy the same positions as the pathologists; they have all other privileges of the medical board, but they are not allowed to vote." At the annual meeting of the medical board of the City Hospital, December, 1901, Dr. Dawbarn moved the said board to grant the present dental staff the privilege to vote, which was carried. At the present time the dental surgeons are enjoying the same privileges as other members of the medical board. This is the first recognition of Dental surgeons in a New York hospital, and it has been brought about through the services of Dr. Dawbarn and his untiring efforts in our behalf, to whom all praise should be given. This recognition of dental surgery as a specialty of medicine is a step forward in our relation and good fellowship with the medical profession.

Very truly yours,

W. C. DEANE.

[The fact may not be generally known that a full staff of dentists has for a year past been connected with the Philadelphia Hospital, and also in addition several young graduates have the appointment as residents.—ED.]

